# Copper Induces Cognitive Impairment in Mice via Modulation of Cuproptosis and CREB Signaling

**DOI:** 10.3390/nu15040972

**Published:** 2023-02-15

**Authors:** Ying Zhang, Qian Zhou, Lu Lu, Yu Su, Wei Shi, Hu Zhang, Ran Liu, Yuepu Pu, Lihong Yin

**Affiliations:** Key Laboratory of Environmental Medicine Engineering, Ministry of Education of China, School of Public Health, Southeast University, Nanjing 210009, China

**Keywords:** Cu, cognitive impairment, cuproptosis, CREB/BDNF

## Abstract

It has been reported that disordered Cu metabolism is associated with several neurodegenerative diseases, including Alzheimer’s disease (AD) and Parkinson’s disease (PD). However, the underlying mechanism is still unclear. In this study, 4-week-old male mice were exposed to Cu by free-drinking water for three months. Then, the effects of Cu on cognitive functions in mice were tested by Morris water maze tests, and the potential mechanisms were investigated by the ELISA, immunochemistry, TUNEL, and Western blot tests. It was found that Cu exacerbates learning and memory impairment, and leads to Cu-overload in the brain and urine of mice. The results showed that Cu induces neuronal degeneration and oxidative damage, promotes the expression of apoptosis-related protein Bax, cuproptosis-related proteins FDX1 and DLAT and the proteotoxic stress marker HSP70, and decreases Fe-S cluster proteins. In addition, Cu affects the pre-synaptic and post-synaptic regulatory mechanisms through inhibiting the expression of PSD-95 and SYP. Cu also suppresses phosphorylation levels in CREB and decreases the expression of BDNF and TrkB in the mouse hippocampus. In conclusion, Cu might mediate cuproptosis, damage synaptic plasticity and inhibit the CREB/BDNF pathway to cause cognitive dysfunction in mice.

## 1. Introduction

The United Nations Environment Programme (UNEP)’s Medium-Term Strategy 2022–2025 states that our planet faces the triple crises of climate change, nature loss and pollution which are jeopardizing global economic and social well-being [1]. The prevention, control and management of pollution are at the heart of improving health, human well-being and prosperity for all. The hemical pollution caused by heavy metals and metalloids is a growing global problem and the main cause of pollution-related diseases in the present world. Long-term exposure to heavy metals may result in adverse health effects, including cardiovascular disease, cancer and altered neuronal function [2,3,4].

Copper (Cu) is one of the most important metals for industrial development. With the increased global demand for Cu products in the past decade, Cu excavation has steadily increased [5]. Cu mining and other industrial activities have contributed to Cu contamination of surface water. It has been reported that Cu ranges from 0.2 to 30 µg/L in freshwater, and reaches an astonishing level of 200,000 µg/L near mining sites [6,7]. With the urbanization and industrialization taking place in China, heavy metal pollution has been found in surface soils and street dusts. It was reported that Cu measured in soil exceeded the baseline levels by several or even dozens of times, and the Cu in street dusts reached up to 623 mg/kg [8]. The health risks caused by environmental Cu pollution require urgent attention.

As one of the essential trace elements in cells, Cu acts as a component of some proteins and nucleic acids, participating in the regulation of life activities [9]. However, an excess of Cu in cells is detrimental and can give rise to several disorders. For example, Cu accumulation in the liver and brain leads to hepatitis and neurodegeneration [10,11]. The number of people suffering from dementia has increased year by year since 1990 and reached 43.8 million worldwide in 2016 [12]. In 2016, dementia was the fifth-largest cause of death globally, and the second-largest cause of death in populations over the age of 70 [12,13]. Alzheimer’s disease (AD) and Parkinson’s disease (PD) are the main causes of dementia in elderly individuals [14,15]. These are neurodegenerative diseases (NDD) with memory loss, progressive cognitive function impairment, behavioral symptoms or functional decline [16,17]. Long-term exposure to a Cu-polluted environment leads to AD, PD and amyotrophic lateral sclerosis (ALS) [18].

Although a variety of neuropathological hallmarks and pathological hypotheses have been proposed, such as neuron deficiencies, oxidative stress, mitochondrial dysfunction, inflammation, abnormal autophagy, accumulation of toxic proteins and cell apoptosis [19], the pathogenesis of neurodegenerative diseases (NDD) remains largely unknown. Neuronal cell death is a direct cause of NDD. Recent studies have shown that the effects on cells induced by copper overload are the same as the cuproptosis induced by copper ionophores [20]. Cuproptosis is a form of Cu-dependent and regulated cell death distinct from known death mechanisms, which provides a new perspective regarding the link between Cu-induced cell death and NDD, advancing our understanding of cell death pathways. Therefore, whether Cu exposure causes neurodegenerative diseases through cuproptosis is worth exploring.

Current research focuses on the health effects of low-level Cu exposure; however, populations in polluted areas generally have higher levels of Cu exposure. Thus, this study aimed to explore the effects of high concentration Cu exposure on learning and memory behavior of C57BL/6J mice to further explain the mechanisms of Cu on cognitive function in mice.

## 2. Materials and Methods

### 2.1. Reagents and Animals

Copper sulfate pentahydrate (CuSO_4_·5H_2_O, purity > 98%, CAS No. 7758-99-8, Sigma-Aldrich, St. Louis, MO, USA) was used for Cu exposure. It was reported that the acute value of Cu exposure ranges from 2.73 µg/L to 107,880 µg/L (~100 mg/L) [21]; thus, copper sulfate pentahydrate was dissolved in distilled water at concentrations of 100 mg/L and 500 mg/L. In order to avoid the influence of the physiological cycle of female mice, thirty C57BL/6J male mice (four-week-old, average 20.20g ± 1.03 g) were purchased from the Weitong Lihua Laboratory Animal Technology Co., Ltd. (Weitong Lihua, Zhejiang, China), experimental animal certificate number is SCXK 2020-0002. The C57BL/6J mice were housed in open-top cages in temperature-controlled rooms at 20~24 °C, with 50–70% relative humidity, a 12 h/12 h light/dark cycle, and ad libitum access to standard rodent chow and distilled water. All the animal studies were approved by the Animal Experimental Ethics of Southeast University (approval ID 20200411003) and conformed with the institutional and National Institute of Health guidelines. Every effort was made to reduce animal suffering and to minimize the number of animals. One of the authors (Y.Z.) received official authorization from the Jiangsu China Experimental Animals Services for animal experimentation.

### 2.2. Experimental Design and Schedule

After a week of acclimatization, the mice were randomly divided into three different groups (N = 10 × 3 = 30): control group (distilled water), low-dose group (100 mg/L Cu), and high-dose group (500 mg/L Cu). The Morris water maze tests were performed after Cu treatment for three months. The exposure mode of free drinking water was simulated. The water in bottles was replaced every two days using 200 mL graduated cylinders. Water and CuSO_4_ solution intakes were measured by measuring cylinder readable to ±0.5 mL. Intakes were read every 24 h (10:00 to 10:00). All the treatments were freshly prepared in distilled water before administration. The mice were weighed every two days.

### 2.3. Morris Water Maze (MWM) Tests

As developed by Morris, MWM was utilized to assess learning and memory ability [22]. The MWM contains a 120 cm-diameter-circular pool and a 50 cm-height platform. The pool tank was filled with opaque water (0.3 m depth) at 22 °C ~ 24 °C. The maze was arbitrarily divided into four equal spaced quadrants (north [N], east [E], south [S], and west [W]). A rounded platform (12 cm in diameter) was hidden in the middle of the NE quadrant (target quadrant), 1.5 cm below the water surface. 

MWM trials consisted of positioning navigation and spatial probe. The positioning navigation test lasted for 4 days, and each mouse was trained 4 times each day. In each session, the mouse was trained to find the platform within 60 s at four different points, and was allowed to stay on the platform for 15 s. If the mouse failed to find the platform within 60 s, it was gently guided to the platform and remained on the platform for 15 s. Four trials were conducted per day with separated intervals of at least 5 min. The amount of time spent finding, climbing the platform (escape latency) and the swimming speed were recorded. The spatial probe test was performed on the last day (day 5). In this test, the platform was removed, and the animals were allowed to explore for 60 s. The time spent in the target platform and quadrant were recorded. All animals were adapted overnight before MWM tests and performed started at 12:00 am. The results were analyzed by Noldus Information Technology (Wageningen, The Netherlands).

At the end of the MWM test, the blood was taken from the eyeball. The mice were euthanized using a ketamine overdose. The brain tissues were immediately removed on ice, either fixed in 4% polyformaldehyde for immunohistochemistry or frozen at −80°C for biochemical analyses.

### 2.4. ICP-MS Assay

#### 2.4.1. Pre-Treatment of Urine Samples

Urine was collected by a metabolic cage for 24 h, 3000× *g* at 4 °C centrifuging for 10 min to remove solids, and urinary creatinine was measured by the automatic biochemical analyzer (Selectra Junior, Deventer, The Netherlands). Then, urine was diluted 10 times with 1% HNO_3_.

#### 2.4.2. Pre-Treatment of Blood Samples

After the MWM test, the blood of the mice was taken from the eyeball, placed at room temperature for about 30 min, centrifuged at 3000 r/min for 5 min and the serum was separated, added with an equal volume of 65% HNO_3_ for nitrification overnight, diluted 20 times with ultra-pure water and centrifuged to obtain the supernatant.

#### 2.4.3. Pre-Treatment of Brain Samples

The mice were sacrificed, the hippocampus tissue was taken in a 15 mL glass tube, 3 mL of 65% HNO_3_ was added and avoided light overnight. The next day, the liquid in the tube was transferred to a small beaker, with 2 mL H_2_O_2_ added and heated on a 200 °C hot plate. When the mixture in the small beaker volatilized to 1 mL, it was removed from the hot plate and returned to room temperature. The content in the small beaker was dissolved with 3 mL 2% HNO_3_ and transferred to a centrifuge tube for collecting the supernatant.

#### 2.4.4. ICP-MS Analysis

ICP-MS (Agilent 7700X, Palo Alto, CA, USA) was used to determine the Cu level in the samples processed above. Urinary Cu was corrected by creatinine, cerebral Cu were calculated using the following formula:

Cerebral Cu content (μg/g) = [measured concentration (μg/L) × 4 mL × 0.001]/organ weight (g).

### 2.5. Hematoxylin-Eosin (HE) Staining

The whole brain was carefully removed at 4 °C and fixed with 4% polyformaldehyde for 24 h. The brain was dehydrated with ethanol, cleared with anhydrous ethanol xylene and embedded in paraffin, then cut into 4 μm sections by paraffin microtome (Leica RM2245, Witzler, Germany). After it was dried, slices were used for HE staining with an automatic staining machine (Leica ST5010, Witzler, Germany). Finally, pathological analysis of the hippocampus was performed by a digital slice scanning system (3 DHISTECH MIDI, Budapest, Hungary).

### 2.6. Immunohistochemical Staining

The brain was collected and fixed in 4% paraformaldehyde at 4 °C overnight and dehydrated in 15% and 30% sucrose. Then, IHC and immunofluorescence were performed to detected expression of NeuN in mice hippocampus. The 4 μm-thick sections were washed with PBS and treated with 0.05% Triton X-100 antigen repair solution. After being blockaded by 5% goat serum for 30 min, the sections were incubated with primary antibody anti-NeuN (Abcam, ab104224, 1:200) overnight at 4 °C and then with biotinylated goat anti-rabbit IgG (Abcam, ab64256, 1:500). After washing, the tissues were incubated in streptavidin-horseradish peroxidase for 1 h at room temperature, incubated in 0.01 m PBS containing 0.05% DAB and 0.003% H_2_O_2_ for 10 min without light, and counterstained with hematoxylin. The localization and distribution of immunoreactive positive materials were acquired on a light microscope (Leica, SP8, Witzler, Germany).

### 2.7. Enzyme-Linked Immunosorbent Assay

The levels of SOD, GPX, MDA (Beyotime Biotechnology, Nanjing, China), dopamine, GABA, and serotonin (5-HT) levels (Jiancheng Bioenineering, Nanjing, China) in the hippocampus were quantified using ELISA kits according to the manufacturer’s procedure. We placed the brain tissue into a 5 mL homogenization tube, added physiological saline at a ratio of weight (g):volume (mL) = 1:9, tissues were homogenized by the tissue grinder at 15,000 rpm/min. We centrifuged the homogenate at 3000 rpm/min for 15 min, then collected supernatant to determine the levels of GPX, SOD, MDA, dopamine, GABA and 5-HT. The absorbance was measured at a wavelength of 450 nm, and the protein concentration was detected according to BCA kits (ThermoFisher, Waltham, MA, USA).

### 2.8. Terminal Deoxynucleotidyl Transferase 2′-Deoxyuridine, 5′-Triphosphate Nick-End Labeling (TUNEL) Assay

TUNEL assay was used for quantification of cell death. Paraffin tissue sections of mice brains were stained by TUNEL (Promega, Madison, WI, USA) and 4′,6-diamidino-2-phenylindole (DAPI, ThermoFisher, Waltham, MA, USA) according to the manufacturer’s instructions. Briefly, the sections mounted on glass slides were deparaffinized and rehydrated. Then, we added 100 μL of proteinase K to each slide and incubated for 15 min at room temperature. Washed with deionized water, added 2% H_2_O_2_ and incubated for 5 min, incubated with TdT equilibration buffer for 10 min and then incubated with TdT enzyme for 1 h in a humidified chamber at 37 °C. We removed the TdT, rinsed in PBS and stained with a DAB staining for 5 min. Washed, dehydrated, cleared and mounted. Images were captured.

### 2.9. Western Blotting

The hippocampal tissues/HT22 cells were homogenized in RIPA buffer with 1% protease and phosphatase cocktail (Beyotime, Nanjing, China). The protein concentration was measured using the BCA protein quantification kit (ThermoFisher, Waltham, MA, USA). A 20 μg protein was separated on 10% SDS-PAGE and then transferred to a PVDF membrane. The membrane was blocked by 5% no-fat milk and then combined with the primary antibody as follows: rabbit-anti-CREB (Abcam, ab178322, 1:1000), rabbit-anti-p-CREB-Ser133 (Abcam, ab32096, 1:1000), mouse-anti-BDNF (proteintech, 66292-1-lg, 1:1000), rabbit-anti-PSD-95 (proteintech, 20665-1-AP, 1:1000), rabbit-anti-SPY (proteintech, 17785-1-AP, 1:1000), rabbit-anti-TrkB (Abclonal, A2099, 1:1000), rabbit-anti-Bax (Abclonal, A19684, 1:1000),rabbit-anti-Bcl-2 (Abclonal, A0208, 1:1000), rabbit-anti-DLAT (Abclonal, A6288, 1:1000), rabbit-anti-DLST (Abclonal, A13297, 1:1000), rabbit-anti-POLD1 (Abclonal, A4218, 1:1000), rabbit-anti-ACO2 (Abclonal, A4524, 1:1000), rabbit-anti-FDX1 (Abclonal, A9815, 1:1000), rabbit-anti-HSP70 (Abclonal, A12948, 1:1000), rabbit-anti-SLC31A1(Abclonal, A0773, 1:1000) and mouse-anti-β-actin (CST, 3700, 1:1000). Membranes were washed and incubated with secondary antibodies goat anti rabbit IgG or goat anti mice IgG (1:5000, Abcam, Cambridge, United Kingdom). Protein expressions were assessed by image J and estimated after normalization calculated by the ratio of the intensity of the band of interest to that of the β-actin.

### 2.10. Cell Line and Treatment

Mouse hippocampal neuronal HT22 cells were provided by the Department of Neurosurgery, Tangdu Hospital, Fourth Military Medical University. The cells were maintained in Dulbecco’s Modified Eagle Medium (DMEM) (Gibco, Gaithersburg, MD, USA) containing 10% fetal bovine serum (FBS) (Gibco, Gaithersburg, MD, USA), 100 U/mL penicillin and 100 mg/L streptomycin (Sigma, St. Louis, MO, USA). Cells were maintained at 37 °C with 5% CO_2_ in a humidified atmosphere. HT22 cells were exposed to 80 μmol/L Cu and Bathocuproinedisulfonic acid or not (BCS, is a known Cu(I)-specific chelating agent [23]) for 48 h. Then, cell apoptosis was detected by flow cytometry and protein expression was detected by Western Blotting. Cell apoptosis was detected using Annexin V-FITC Apoptosis Detection Kit (KeyGEN, Nanjing, China) according to the protocol.

Lentivirus expressing CREB/negative control (NC) (lentivirus-CREB and lentivirus-NC (ViGene Biosciences, Shandong, China) were transfected into HT22 cells. The plasmid splicing sequence was was shown in Supplementary Text, lentiviral vector of CREB gene was shown in Supplementary Figure S1. Firstly, 3 × 10^4^ cells were seeded in 24-well plates, then 15 mL each of Lv-CREB lentivirus and LV-NC lentivirus (1 × 10^8^ transducing units (TU)/mL, MOI = 50) as well as 8 mg/mL infection reagent ADV-HR diluted by DMEM was added. Incubated for 12 h, the medium was refreshed, and continued to be incubated for 24–96 h. Transfection efficiency was determined by a fluorescence microscope (AX10, ZEISS, Oberkochen, Germany) and Western Blotting. Lv-CREB-HT22 and Lv-NC-HT22 cells were treated with 80 μmoL Cu, and then cells were collected to conduct apoptosis assay and Western blotting. Apoptosis of LV-CREB and LV-NC HT22 cell was detected using Annexin V-PE/7-AAD Apoptosis Detection Kit (Yeasen Biotechnology, Shanghai, China) according to the protocol.

### 2.11. Data Analysis

SPSS 20.0 and the GraphPad Prism 9 software were used for data analysis. Wb and TUNEL results were processed using ImageJ software (RRID: SCR_003070). Data were listed as means ± SD, and analyzed through ANOVA analysis and Dunnett’s multiple comparisons test. Differences between groups were considered to be statistically significant at *p* < 0.05 (*), *p* < 0.01 (**) and *p* < 0.001 (***).

## 3. Results

### 3.1. Cu Induces Spatial Reference Memory Impairment Impair of C57BL/6J Mice

**Figure 1:**
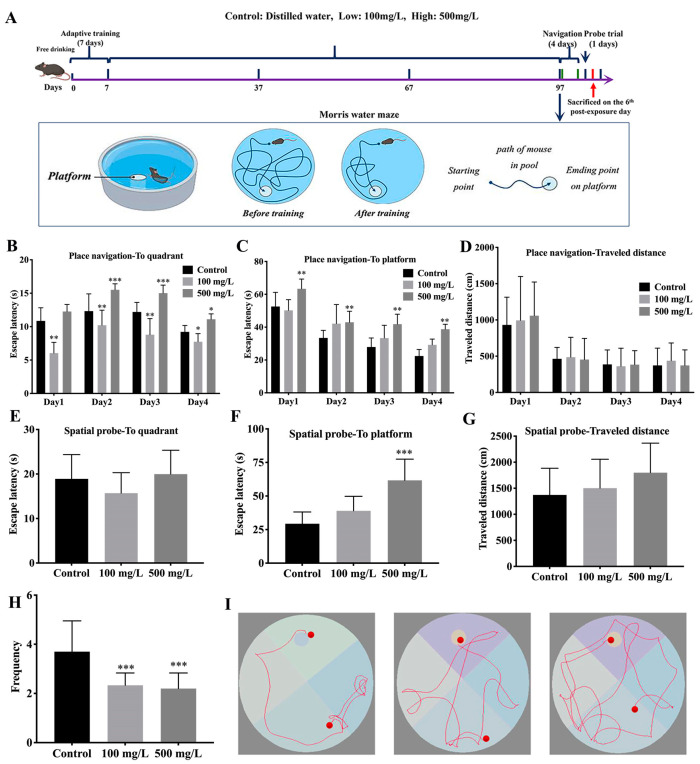
Cu impairs learning and memory in mice. (**A**) Schematic of study protocol timeline. After one-week acclimatization, the mice were induced for 90 consecutive days. After 97 days, MWM tests were performed. Towards the end of the tests, the mice were sacrificed by anaesthetization. MWM: Morris water maze, total N = 10 × 3 = 30. (**B**) Escape latency in the target quadrant. (**C**) Escape latency in the target platform. (**D**) Travel distance in the spatial training. (**E**) The latency of target quadrant in the probe trial. (**F**) The latency of target platform in the probe trial. (**G**) Travel distance in the probe trial. (**H**) The crossing-platform times in the probe trial. (**I**) Representative tracking paths of mouse in MWM. (* *p* < 0.05, ** *p* < 0.01, *** *p* < 0.001).

There was no animal that died during the whole experiment. The mice reacted quickly and positively. Figure 1A shows the schematic study protocol timeline. To evaluate the effects of Cu on cognitive function in the C57BL/6J mice, the Morris water maze test was performed. Figure 1I showed the tracks of mice in the maze. In the 4-day navigation test, Cu increased the target quadrant latency and the platform latency of mice in the 500 mg/L group (Figure 1B,C, * *p* < 0.05, ** *p* < 0.01, *** *p* < 0.001), but not travel distance (Figure 1D). On the probe trial day, Cu prolonged platform latency (Figure 1F, *** *p* < 0.001) but not target quadrant latency and travel distance (Figure 1E,G). In addition, Cu degraded the frequency of crossing-platform (Figure 1H, *** *p* < 0.001). These results indicate that Cu could impair cognitive function in mice.

### 3.2. Cu Exposure Increases Brain Cu Levels and Damages Hippocampal Tissue of C57BL/6J Mice

**Figure 2:**
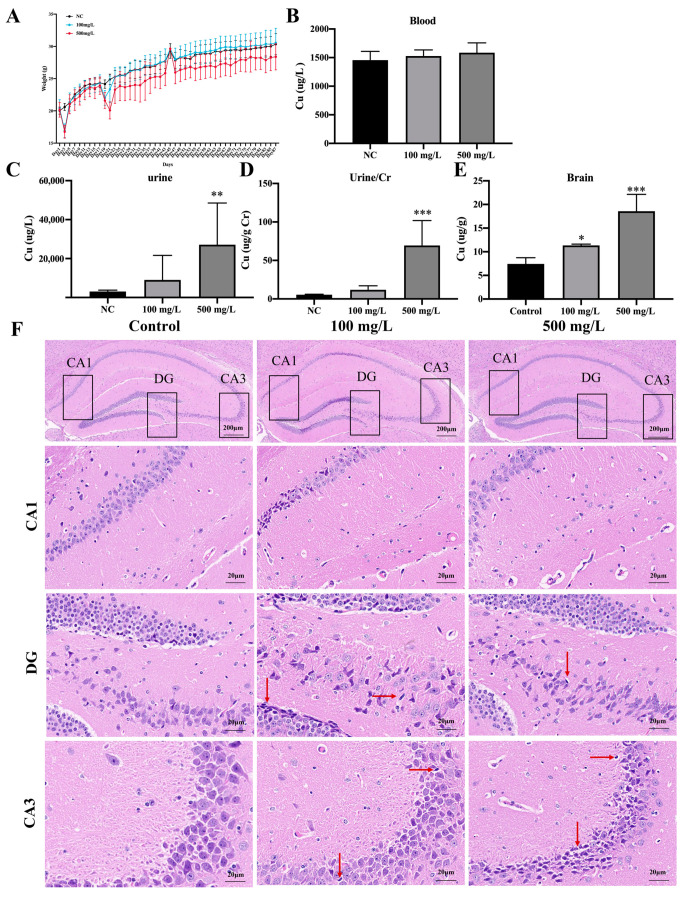
Cu exposure induces Cu accumulation and neuronal degeneration in mice hippocampus. (**A**) The body weight of the mice. (**B**) Cu level in blood of mice. (**C**) Cu level in urine of mice. (**D**) Creatinine-corrected Cu level in urine of mice. (**E**) Cu level in hippocampus tissue of mice. (**F**) HE staining of mice hippocampus after Cu exposure. Red arrows showed cells arranged in a disorderly manner, cytoplasm and nucleolus deeply dyed with nuclear pyknosis. Scale bar = 200 μm in first row, scale bar = 20 μm in figure of CA1, CA3 and DG regions. * *p* < 0.05, ** *p* < 0.01, *** *p* < 0.001.

During the period of acclimatization, we found that the average daily water consumption of mice was 5 mL, and the average daily consumption of Cu-containing water was 2~3 mL. To obtain the actual Cu intake of mice, the average Cu intake of mice was calculated based on the daily water intake (Table 1, Figures S2 and S3). The daily water intake of mice in each group was stable. Compared with the control group, the bodyweight of the mice decreased significantly in the 500 mg/L Cu group (Figure 2A). As Table 1 shows, the average daily and Cu intake of unit weight mice were promoted with increasing Cu exposure (*** *p* < 0.001).

After Cu exposure, ICP-MS was performed to detect Cu levels in the urine, serum and brain tissues of mice. The results showed urinary Cu was increased especially in the 500 mg/L group (Figure 2C, ** *p* < 0.01). In addition, Cu levels were increased after being corrected by creatinine (Figure 2D, *** *p* < 0.001). However, serum Cu in mice was increased slightly (Figure 2B). Furthermore, the results showed that the Cu levels in hippocampus tissue were promoted significantly in a dose-dependent manner (Figure 2E, * *p* < 0.05, *** *p* < 0.001).

Figure 2F showed representative images of HE staining in hippocampus tissues. The neurons were arranged neatly and tightly in hippocampus of control mice, while the neurons of Cu-exposed mouse showed the pyknotic and hyperchromatic, pyramidal neuron cell necrosis, as well as more extensive karyopyknosis and karyolysis (indicated by the red arrow).

### 3.3. Cu Induces Oxidative Damage and Promotes Apoptosis in Hippocampal Tissue

**Figure 3:**
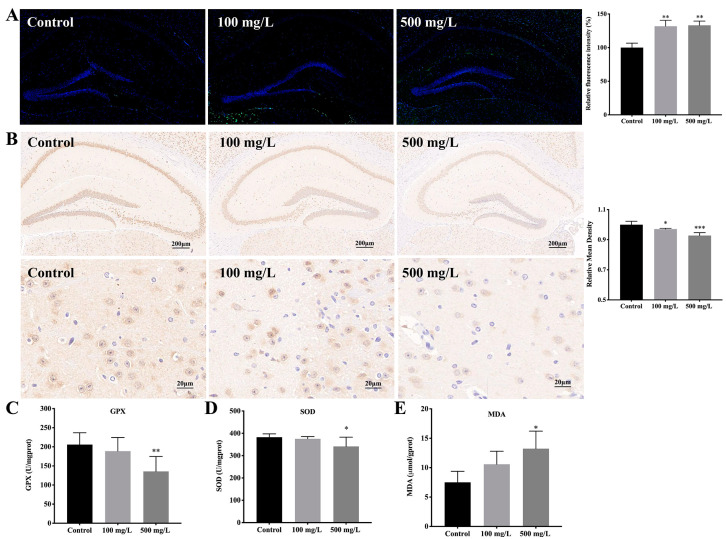
Cu induces oxidative damage and promotes apoptosis in mice hippocampal. (**A**) TUNEL staining of mice hippocampus after Cu exposure and relative fluorescence intensity of TUNEL-positive cells, green color indicates TUNEL-positive cells and blue indicates DAPI-stained nucleus, scale bar = 200 μm. (**B**) The effect of Cu exposure on the expression of NeuN and relative mean density of NeuN-positive cells. NeuN: neuron-specific nuclear proteins. Scale bar = 200 μm in first row in B, scale bar = 20 μm in second row in B. (**C**–**E**) GPX, SOD and MDA levels in the brain of the mice. * *p* < 0.05, ** *p* < 0.01, *** *p* < 0.001.

Figure 3A showed the results of apoptosis, the green fluorescence intensity in mouse hippocampus was increased with Cu treatment (Figure 3A,B, ** *p* < 0.01). The neuronal loss was further measured by the neuronal nuclear protein (NeuN). As shown in Figure 3C, the expression of NeuN was lower in the brains of Cu-exposed mice compared with the control mice (Figure 3C,D, * *p* < 0.05, *** *p *< 0.001). 

Our previous in vitro studies found that Cu promoted the apoptosis of HT22 cells through oxidative stress [23]. Thus, indicators of cellular oxidative stress in mice brains were detected. The results showed that Cu significantly reduced the activity of GPX and the activity of SOD and elevated the level of MDA in the mouse brain (Figure 3C–E, * *p* < 0.05, ** *p* < 0.01).

### 3.4. Cu Inhibits CREB/BDNF Signaling Pathway

**Figure 4:**
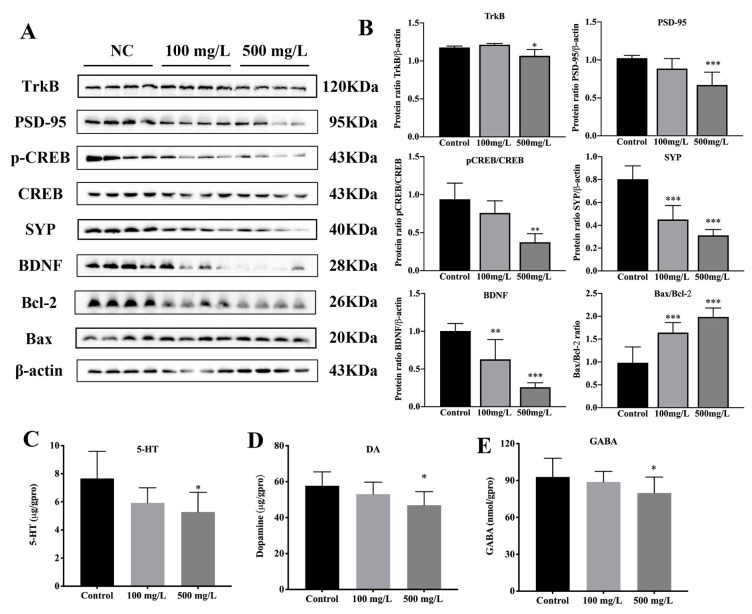
Cu impairs CREB/BDNF signaling of mice hippocampal. (**A**) Effects of Cu on the proteins expression of TrkB, PSD-95, pCREB, CREB, SYP, BDNF and Bax, Bcl-2 in mice hippocampus. (**B**) Quantitative analysis of TrkB, PSD-95, pCREB/CREB, SYP, BDNF and Bax/Bcl-2. (**C**–**E**) Serotonin (5-HT), dopamine (DA), and γ-aminobutyric acid (GABA) levels in the brain of the mice. * *p* < 0.05, ** *p* < 0.01, *** *p* < 0.001.

To explore whether Cu exposure affects the CREB/BDNF signaling pathway, the proteins of CREB/BDNF signaling pathway were measured by Western blot analysis. The expression of CREB showed no significant difference among the three groups, while phosphorylation of CREB was reduced by Cu (Figure 4A,B, ** *p* < 0.01). The expression of BDNF was significantly inhibited by Cu (Figure 4A,B, ** *p* < 0.01, *** *p* < 0.001). In addition, Cu inhibited the expressions of TrkB, PSD-95 and SYP in a dose-dependent manner (Figure 4A,B, * *p* < 0.05, *** *p* < 0.001). The apoptosis protein Bax was up-regulated and the anti-apoptosis protein Bcl-2 was down-regulated with Cu treatment; in particular, the Bax/Bcl-2 ratio was increased (Figure 4A,B, *** *p* < 0.001). Neurotransmitters levels including Serotonin (5-HT), dopamine (DA) and γ-aminobutyric acid (GABA) in mouse brain tissue were reduced by Cu (Figure 4C–E, * *p* < 0.05).

**Figure 5:**
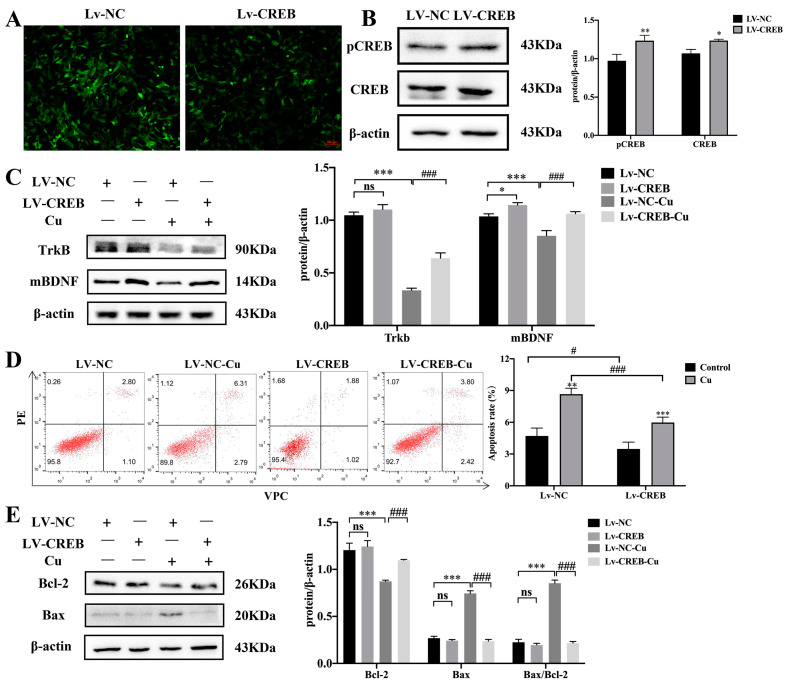
Effects of Cu on the CREB-upregulated HT22 cells. (**A**) HT22 cells transfected with lentivirus were pictured by fluorescence microscope (scale bar = 200 μm). (**B**) The protein expression and quantitative analysis of pCREB and CREB after LV-CREB transfection. (**C**) The protein expression and quantification of TrkB and mBDNF after LV-CREB and Cu treatment. (**D**) Flow cytometry results and apoptotic index of HT22 cells after LV-CREB and Cu treatment. (**E**) The protein expression and quantification of Bax and Bcl-2 after LV-CREB and Cu treatment. * *p* < 0.05, ** *p* < 0.01, *** *p* < 0.001, compared with LV-NC; # *p* < 0.05, ### *p* < 0.001, compared with LV-NC-Cu; ns means no significant difference.

To further explore the role of CREB during Cu exposure, HT22 cells were infected with lentivirus to up-regulate the expression of CREB (Figure 5A). The protein levels of pCREB and CREB in HT22 cells were increased with viral infection (Figure 5A,B, * *p* < 0.05, ** *p* < 0.01). Furthermore, Lv-CREB-HT22 and Lv-NC-HT22 cells were treated with Cu. The results showed that the expression of mBDNF and TrkB were promoted by CREB, and CREB partially rescued the inhibition of mBDNF and TrkB induced by Cu (Figure 5C, * *p* < 0.05, *** *p* < 0.001, ### *p* < 0.001). Interestingly, CREB could prevent Cu-related apoptosis, the expression of apoptosis-related proteins Bax and anti-apoptosis protein Bcl-2 were the opposite of the Cu-treated HT22 cells (Figure 5D,E, ** *p* < 0.01, *** *p* < 0.001, # *p* < 0.05, ### *p* < 0.001).

### 3.5. Cu Induces Cuproptosis

**Figure 6:**
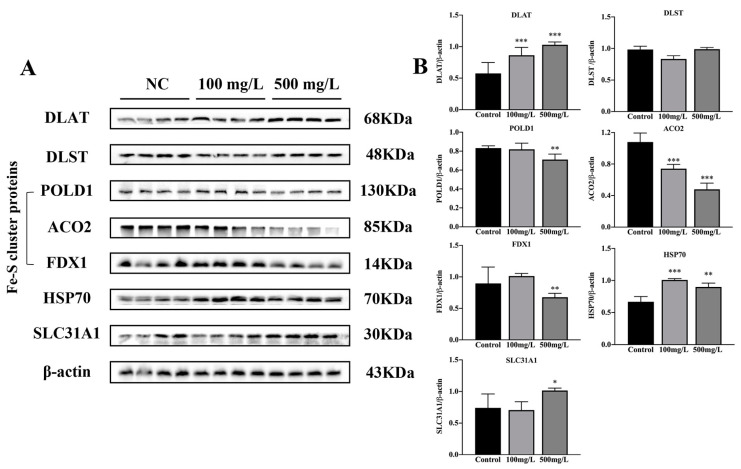
Effects of Cu on the expression of cuproptosis-related protein. (**A**) Proteins expression of DLAT, DLST, POLD1, ACO2, FDX1, HSP70 and SLC31A1 in mouse hippocampus. (**B**) Quantitative analysis of DLAT, DLST, POLD1, ACO2, FDX1, HSP70 and SLC31A1, * *p* < 0.05, ** *p* < 0.01, *** *p* < 0.001.

With Cu exposure, the protein lipoylation enzyme DLAT was observed to increase in the dose-dependent manner (Figure 6, *** *p* < 0.001). The expressions of Fe-S cluster proteins, FDX1, POLD1 and ACO2 were inhibited by Cu (Figure 6, ** *p* < 0.01, *** *p* < 0.001). In addition, the Cu importer SLC31A1 (CTR1) and proteotoxic stress marker HSP70 were increased with Cu exposure (Figure 6, * *p *< 0.05, ** *p* < 0.01, *** *p* < 0.001).

**Figure 7:**
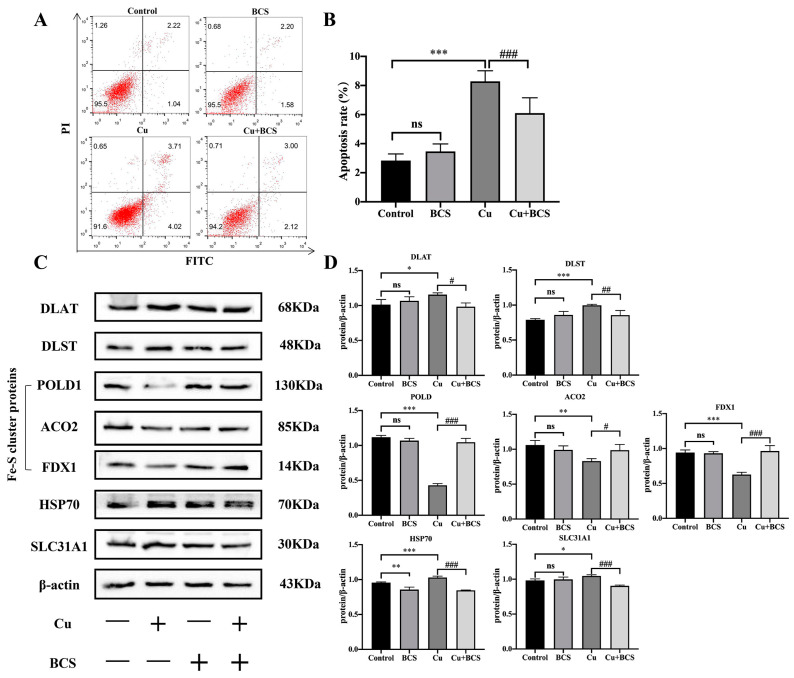
BCS rescues copper-induced neuronal apoptosis. (**A**,**B**) Flow cytometry results and apoptotic index of HT22 cells after Cu and BCS treatment. (**C**,**D**) The protein expression and quantification of DLAT, DLST, POLD1, ACO2, FDX1, HSP70 and SLC31A1 after being treated with Cu as well as BCS. * *p* < 0.05, ** *p* < 0.01, *** *p* < 0.001, compared with control group; # *p* < 0.05, ## *p* < 0.01, ### *p* < 0.001, compared with Cu-treated group; ns means no significant difference.

Our previous study found that Cu promoted the apoptosis of HT22 cells [24]. To clarify the relationship between Cu and cuproptosis, BCS was used to remove Cu. Cells were treated with Cu (80 μmol/L) alone or in combination with BCS. BCS is a known Cu(I)-specific chelating agent [23]. As Figure 6A shows, BCS could partially rescue Cu-induced apoptosis (Figure 7A,B, * *p* < 0.05, *** *p* < 0.001). The increased levels of DLAT and DLST induced by Cu were dropped by BCS. The Fe-S cluster proteins POLD1, POLD1 and FDX1 were restored by BCS. The expression of CTR1 and HSP70 were down-regulated after removing Cu by BCS (Figure 7C,D, compared with control: * *p* < 0.05, ** *p* < 0.01, *** *p* < 0.001, compared with Cu: # *p* < 0.05, ## *p* < 0.01, ### *p* < 0.001).

## 4. Discussion

### 4.1. Cu Might Induce Cognitive Impairment in Mice via Modulation of Cuproptosis

The primary sources of Cu are from Cu water pipes, Cu cookware, electronic waste, drinking water, medicines, dietary supplements and Cu-containing fungicides [25]. Drinking water is the main source of human non-occupational Cu exposure. In this study, drinking water was used for Copper exposure, which is closer to the exposure manner of humans than gavage or intraperitoneal injection. Armstrong [26] found neuronal death after injecting cupric sulfate into the hippocampus of rats. Zhang [27] revealed that the intraperitoneal injection of CuCl_2_ in SD rats led to decreased spatial learning and memory. In the present study, the ICP-MS results showed that excessive intake of Cu, on the one hand, increased the Cu excretion of renal and, on the other hand, promoted the Cu accumulation in the hippocampus of mice. In order to demonstrate the effect of Cu on the learning and memory of mice and its possible mechanism, MWM tests, in vivo and in vitro experiments were conducted. Studies have shown that Cu overload in the hippocampus was related to the deletion and dysfunction of neuron cells. Furthermore, the positioning navigation results showed that Cu increased the escape latency of platform and the target quarter. The probe trial results showed that the platform latency of the mice was prolonged and crossing-platform times were reduced by Cu. These results suggested Cu exposure induced learning and memory impairment. 

One of the important explanations of brain damage caused by the dysregulation of Cu homeostasis is Cu cycles between of its oxidative states (Cu^2+^ and Cu^+^). The conversion of Cu^2+^/Cu^+^ oxidation state and reduction state cycling system induces Fenton and Haber–Weiss reactions, producing ROS products such as hydrogen peroxide (H_2_O_2_), superoxide radical (•O_2−_) and hydroxyl radical (•OH) [28]. As an intermediate product of the oxidation-reduction reaction, ROS is also produced from normal cell metabolism. However, the over-production of ROS will attack biomacromolecules, contributing to oxidative damage to proteome, lipids and DNA [29]. ROS products attacking intracellular biomacromolecules are the precursors of Cu-induced neurotoxicity and cell dysfunction [30]. Antioxidant defenses, including enzyme antioxidant system and non-enzyme antioxidant system, play an important role in fighting oxidative stress [31]. Enzymatic antioxidants include superoxide dismutase (SOD), catalase (CAT), glutathione peroxidase (GSH-Px, GPX) and glutathione reductase (GR) [32]. In this study, the expressions of SOD and GPX in the brain of mice exposed to Cu were gradually inhibited, suggesting that Cu induced oxidative damage in the brains of mice. In addition, the lipid peroxide malondialdehyde (MDA) was increased, suggesting that Cu exposure led to lipid damage.

Neuronal loss contributes to memory deficits and cognitive impairment [33,34]. Thus, we observed the effect of Cu on neuronal apoptosis. Firstly, HE staining revealed that pathological damage in the hippocampus was caused by excessive Cu. TUNEL staining suggested the neuronal apoptosis of the hippocampus was increased after Cu exposure. The neuronal nuclear marker (NeuN) is a well-recognized marker for mature neurons, whose expression level has been used to assess neuronal death [35]. In light of the results of NeuN antibody labeling, we considered that Cu induced the loss of neurons and promoted neuronal apoptosis in the hippocampus of mice. TSVETKOV proposed that Cu overload led to a unique type of copper-induced regulated cell death, named “cuproptosis”, which was distinct from all other known mechanisms of regulated cell death [20]. The characteristics of Cu-dependent death are lipoylated protein aggregation and subsequent iron-sulfur (Fe-S) cluster protein loss, which leads to proteotoxic stress and, ultimately, cell death [20]. Dihydrolipoamide S-succinyltransferase (DLST) and dihydrolipoamide S-acetyltransferase (DLAT) were enzymes regulating protein lipoylation [36]. Our results showed that the Cu importer SLC31A1/CTR1 was up-regulated to promote the transportation of Cu. DLAT was increased and the Fe-S cluster, including FDX1, ACO2 and POLD1, was inhibited by Cu, and the expression of HSP70 was increased. Furthermore, changes in the level of apoptosis and the expression of cuproptosis-related proteins in HT22 cells were partially restored after Cu removal by BCS, suggesting that the death of neuronal cells caused by Cu may be mediated by cuproptosis. However, whether cuproptosis contributes to Cu-induced cognitive impairment remains to be determined through more work.

### 4.2. Cu Destroys Synaptic Plasticity and CREB-Mediated Memory Formation 

Studies have shown that hippocampal dysfunction occurred in the early stage of AD, accompanied by long-term potentiation (LTP) inhibition and synaptic dysfunction in the patient’s brain [37]. Synaptic dysfunction has also been observed in other neurodegenerative diseases, such as PD and Huntington’s disease (HD) [38]. The synapse includes a presynaptic terminal, synaptic cleft and a postsynaptic compartment, comprising the unit for electrical activity transformation between neural cells [39]. Synaptic plasticity is manifested as adaptive changes in synaptic morphology and function, including long-term potentiation (LTP) and long-term inhibition (LTD). The coordination of the two changes is the neurophysiological basis for the formation of learning and memory [40]. Studies have shown that both the presynaptic mechanism and the postsynaptic mechanism participated in the regulation of synaptic plasticity [40]. Expression of SYP is closely related to the number of presynaptic vesicles, whose density and distribution indirectly reflected the number and distribution of synapses [41]. Postsynaptic compact protein-95 (PSD-95) plays a role in the supporting and anchoring of postsynaptic receptors. It is involved in advanced brain function, such as learning and the memory of cortical circuits, by regulating the organization of receptors and related proteins [42]. It was found that the expression of SYP and PSD-95 were significantly reduced in the hippocampus of memory deficits in mice [43,44]. Transmitters in synapses and neuronal sites, including dopamine, 5-HT and GABA, are closely related to synaptic strength and modulation [45,46]. Moreover, dysregulation of dopamine, 5-HT and GABA might modulate and affect learning and memory [45,46]. In our study, Cu exposure reduced the expression of SYP, PSD-95 and transmitters including dopamine, 5-HT and GABA in mice, suggesting that Cu simultaneously affects the pre-synaptic regulatory mechanisms, post-synaptic regulatory mechanisms and release of transmitters. A decreased level of SYP inhibited the transport capacity of synaptic vesicles and the nervous system’s information transmission, processing and storage. In addition, Cu reduced post-synaptic dense substances or synaptic activity, leading to obstacles in synaptic conduction.

Apart from the modification of synaptic plasticity, CREB-mediated changes play an indispensable role in memory formation [47]. Brain-derived neurotrophic factor (BDNF) is one of prominent targets of CREB, interestingly, it can also modulate its function by mediating CREB transcription factor. The transcription factor CREB initiates the expression of BDNF and then BNDF binds to its receptor TrkB to activate CREB [48,49], forming a feedback loop. BDNF is a regulator of synaptic plasticity and is also involved in neuroprotection and neuroregeneration [50]. It is proposed that the derangement of CREB phosphorylation and the transcription machinery interacting with CREB play a key role in synaptic dysfunction and memory loss [51,52]. In addition, other signals such as the ERK1/2 cascades, Ca^2+^ and 5-HT could also activate CREB [52,53]. Our previous study found oxidative damage could inhibit the activity of CREB [24]. Thus, Cu-activated oxidative stress might reduce phosphorylation levels of CREB and then inhibit the translocation of CREB to decrease the expression of the downstream proteins BDNF and TrkB. In addition, decreased 5-HT levels also prevented the activation of CREB. Most importantly, CREB prevents HT22 cells from apoptosis induced by Cu.

Therefore, Cu might arrest BDNF to inhibit synaptic plasticity through CREB/BDNF pathways, contributing to impaired neuroprotection and impediment to memory activation and consolidation. Furthermore, Cu also promotes the apoptosis of neurons in the hippocampus, resulting in an abnormal neuronal circuit. However, the specific regulatory mechanism still requires research evidence to provide further confirmation, and would provide a basis for the treatment of Cu-induced neurodegenerative diseases.

## 5. Conclusions

Our study demonstrates that excessive Cu exposure leads to Cu accumulation in mouse brain tissue and induces learning and memory impairment in mice. Further studies show that Cu overload induces the pathological injury of brain tissue through oxidative damage and cell apoptosis. Mechanism studies prove that Cu impairs synaptic plasticity through CREB/BDNF pathways and stimulates cuproptosis to promote cell death.

## Figures and Tables

**Table 1:** Analysis of water and Cu intake in mice.

Group	Control	100 mg/L	500 mg/L
Water intake (mL)	5.112 ± 0.712	4.072 ± 0.882 ^a^	2.817 ± 0.836 ^ab^
Daily Cu intake (mg/mouse)	0	0.407 ± 0.083 ^a^	1.409 ± 0.392 ^ab^
Cu intake of unit weight (mg/g)	0	0.015 ± 0.003 ^a^	0.055 ± 0.016 ^ab^

^a^: *p* < 0.001, compared to the control, ^b^: *p* < 0.001, compared to the 100 mg/L group.

## Data Availability

Not applicable.

## Supplementary Materials

The following supporting information can be downloaded at: https://www.mdpi.com/article/10.3390/nu15040972/s1, Supplementary Text Plasmid splicing sequence, Figure S1 Lentiviral vector of mouse CREB gene overexpression, Figure S2 Water intake of the mice, Figure S3 Copper intake of the mice.

## References

[1] Programme U.E. (2021). Medium-Term Strategy 2022–2025. The United Nations Environment Programme Strategy for Tackling Climate Change, Biodiversity and Nature Loss, and Pollution and Waste from 2022–2025.

[2] Cosselman K.E., Navas-Acien A., Kaufman J.D. (2015). Environmental factors in cardiovascular disease. Nat. Rev. Cardiol..

[3] Tanvir E.M., Komarova T., Comino E., Sumner R., Whitfield K.M., Shaw P.N. (2021). Effects of storage conditions on the stability and distribution of clinical trace elements in whole blood and plasma: Application of ICP-MS. J. Trace Elem. Med. Biol..

[4] Yang F., Yi X., Guo J., Xu S., Xiao Y., Huang X., Duan Y., Luo D., Xiao S., Huang Z. (2019). Association of plasma and urine metals levels with kidney function: A population-based cross-sectional study in China. Chemosphere.

[5] Izydorczyk G., Mikula K., Skrzypczak D., Moustakas K., Witek-Krowiak A., Chojnacka K. (2021). Potential environmental pollution from copper metallurgy and methods of management. Environ. Res..

[6] Flemming C.A., Trevors J.T. (1989). Copper Toxicity and Chemistry in the Environment—A review. Water Air Soil Pollut..

[7] Davis A., Ashenberg D. (1989). The aqueous geochemistry of the Berkeley Pit, Butte, Montana, USA. Appl. Geochem..

[8] Wei X., Gao B., Wang P., Zhou H., Lu J. (2015). Pollution characteristics and health risk assessment of heavy metals in street dusts from different functional areas in Beijing, China. Ecotoxicol. Environ. Saf..

[9] Nose Y., Kim B.E., Thiele D.J. (2006). Ctr1 drives intestinal copper absorption and is essential for growth, iron metabolism, and neonatal cardiac function. Cell Metab..

[10] Chandan V.S., Shah S.S., Mounajjed T., Torbenson M.S., Wu T.-T. (2017). Copper deposition in focal nodular hyperplasia and inflammatory hepatocellular adenoma. J. Clin. Pathol..

[11] Gromadzka G., Tarnacka B., Flaga A., Adamczyk A. (2020). Copper Dyshomeostasis in Neurodegenerative Diseases—Therapeutic Implications. Int. J. Mol. Sci..

[12] Feigin V.L., Nichols E., Alam T., Bannick M.S., Beghi E., Blake N., Culpepper W.J., Dorsey E.R., Elbaz A., Ellenbogen R.G. (2019). Global, regional, and national burden of neurological disorders, 1990–2016: A systematic analysis for the Global Burden of Disease Study 2016. Lancet Neurol..

[13] Naghavi M., Abajobir A.A., Abbafati C., Abbas K.M., Abd-Allah F., Abera S.F., Aboyans V., Adetokunboh O., Afshin A., Agrawal A. (2017). Global, regional, and national age-sex specific mortality for 264 causes of death, 1980–2016: A systematic analysis for the Global Burden of Disease Study 2016. Lancet.

[14] Atri A. (2019). The Alzheimer’s Disease Clinical Spectrum: Diagnosis and Management. Med. Clin. North Am..

[15] Goldman J.G., Sieg E. (2020). Cognitive Impairment and Dementia in Parkinson Disease. Clin. Geriatr. Med..

[16] Cui M.Y., Lin Y., Sheng J.Y., Zhang X., Cui R.J. (2018). Exercise Intervention Associated with Cognitive Improvement in Alzheimer’s Disease. Neural Plast..

[17] Lawson R.A., Yarnall A.J., Duncan G.W., Breen D.P., Khoo T.K., Williams-Gray C.H., Barker R.A., Collerton D., Taylor J.-P., Burn D.J. (2016). Cognitive decline and quality of life in incident Parkinson’s disease: The role of attention. Park. Relat. Disord..

[18] Scheiber I., Dringen R., Mercer J.F. (2013). Copper: Effects of deficiency and overload. Met. Ions. Life Sci..

[19] Grewal A.K., Singh T.G., Sharma D., Sharma V., Singh M., Rahman H., Najda A., Walasek-Janusz M., Kamel M., Albadrani G.M. (2021). Mechanistic insights and perspectives involved in neuroprotective action of quercetin. Biomed. Pharmacother..

[20] Tsvetkov P., Coy S., Petrova B., Dreishpoon M., Verma A., Abdusamad M., Rossen J., Joesch-Cohen L., Humeidi R., Spangler R.D. (2022). Copper induces cell death by targeting lipoylated TCA cycle proteins. Science.

[21] USEPA (2007). Aquatic Life Ambient Freshwater Quality Criteria-Copper.

[22] Morris R.G.M., Garrud P., Rawlins J.N.P., O’Keefe J. (1982). Place navigation impaired in rats with hippocampal lesions. Nature.

[23] Li Y., Trush M.A. (1993). DNA damage resulting from the oxidation of hydroquinone by copper: Role for a Cu(II)/Cu(I) redox cycle and reactive oxygen generation. Carcinogenesis.

[24] Lu Q., Zhang Y., Zhao C., Zhang H., Pu Y., Yin L. (2021). Copper induces oxidative stress and apoptosis of hippocampal neuron via pCREB/BDNF/ and Nrf2/HO-1/NQO1 pathway. J. Appl. Toxicol..

[25] Bost M., Houdart S., Oberli M., Kalonji E., Huneau J.-F., Margaritis I. (2016). Dietary copper and human health: Current evidence and unresolved issues. J. Trace Elements Med. Biol..

[26] Armstrong C., Leong W., Lees G.J. (2001). Comparative effects of metal chelating agents on the neuronal cytotoxicity induced by copper (Cu^+2^), iron (Fe^+3^) and zinc in the hippocampus. Brain. Res..

[27] Zhang G., Li Q., Gao W., Liu S., Wu R., Shen Z., Liu W., Chen Y. (2017). Copper chloride dose-dependently alters spatial learning and memory, and glutamate levels, in the hippocampus of rats. Mol. Med. Rep..

[28] Halliwell B., Gutteridge J.M. (1990). Role of free radicals and catalytic metal ions in human disease: An overview. Methods Enzym..

[29] Pisoschi A.M., Pop A., Iordache F., Stanca L., Predoi G., Serban A.I. (2020). Oxidative stress mitigation by antioxidants—An overview on their chemistry and influences on health status. Eur. J. Med. Chem..

[30] Turgut G., Akdogan I., Adiguzel E., Genç O. (2003). Effect of Copper Overload Together with Ethanol Uptake on Hippocampal Neurons. Tohoku J. Exp. Med..

[31] Indo H.P., Yen H.-C., Nakanishi I., Matsumoto K.-I., Tamura M., Nagano Y., Matsui H., Gusev O., Cornette R., Okuda T. (2015). A mitochondrial superoxide theory for oxidative stress diseases and aging. J. Clin. Biochem. Nutr..

[32] Rochette L., Lorin J., Zeller M., Guilland J.-C., Lorgis L., Cottin Y., Vergely C. (2013). Nitric oxide synthase inhibition and oxidative stress in cardiovascular diseases: Possible therapeutic targets?. Pharmacol. Ther..

[33] Nobili A., Latagliata E.C., Viscomi M.T., Cavallucci V., Cutuli D., Giacovazzo G., Krashia P., Rizzo F.R., Marino R., Federici M. (2017). Dopamine neuronal loss contributes to memory and reward dysfunction in a model of Alzheimer’s disease. Nat. Commun..

[34] Yan W., Fan J., Zhang X., Song H., Wan R., Wang W., Yin Y. (2021). Decreased neuronal synaptosome associated protein 29 contributes to poststroke cognitive impairment by disrupting presynaptic maintenance. Theranostics.

[35] Duan W., Zhang Y.-P., Hou Z., Huang C., Zhu H., Zhang C.Q., Yin Q. (2016). Novel Insights into NeuN: From Neuronal Marker to Splicing Regulator. Mol. Neurobiol..

[36] Rowland E.A., Snowden C.K., Cristea I.M. (2018). Protein lipoylation: An evolutionarily conserved metabolic regulator of health and disease. Curr. Opin. Chem. Biol..

[37] Sánchez-Rodríguez I., Gruart A., Delgado-García J.M., Jiménez-Díaz L., Navarro-López J.D. (2019). Role of GirK Channels in Long-Term Potentiation of Synaptic Inhibition in an In Vivo Mouse Model of Early Amyloid-β Pathology. Int. J. Mol. Sci..

[38] Taoufik E., Kouroupi G., Zygogianni O., Matsas R. (2018). Synaptic dysfunction in neurodegenerative and neurodevelopmental diseases: An overview of induced pluripotent stem-cell-based disease models. Open Biol..

[39] Ilic K., Mlinac-Jerkovic K., Sedmak G., Rosenzweig I., Kalanj-Bognar S. (2021). Neuroplastin in human cognition: Review of literature and future perspectives. Transl. Psychiatry.

[40] Wang H., Xu X.X., Xu X.X., Gao J., Zhang T. (2020). Enriched Environment and Social Isolation Affect Cognition Ability via Altering Excitatory and Inhibitory Synaptic Density in Mice Hippocampus. Neurochem. Res..

[41] Zhu X.K., Wang P., Liu H.J., Zhan J., Wang J., Li M., Zeng L., Xu P. (2019). Changes and Significance of SYP and GAP-43 Expression in the Hippocampus of CIH Rats. Int. J. Med. Sci..

[42] Pollak D.D., Herkner K., Hoeger H., Lubec G. (2005). Behavioral testing upregulates pCaMKII, BDNF, PSD-95 and egr-1 in hippocampus of FVB/N mice. Behav. Brain Res..

[43] Wang D.D., Li B., Wu Y.P., Li B. (2019). The Effects of Maternal Atrazine Exposure and Swimming Training on Spatial Learning Memory and Hippocampal Morphology in Offspring Male Rats via PSD95/NR2B Signaling Pathway. Cell. Mol. Neurobiol..

[44] Liu Y., Zhang X., Yan D.D., Wang Y., Wang N., Liu Y., Tan A., Chen X., Yan H. (2020). Chronic acrylamide exposure induced glia cell activation, NLRP3 inflammasome upregulation and cognitive impairment. Toxicol. Appl. Pharmacol..

[45] Marinesco S., Carew T.J. (2002). Serotonin release evoked by tail nerve stimulation in the CNS of aplysia: Characterization and relationship to heterosynaptic plasticity. J. Neurosci..

[46] Ben-Ari Y., Gaiarsa J.L., Tyzio R., Khazipov R. (2007). GABA: A pioneer transmitter that excites immature neurons and generates primitive oscillations. Physiol. Rev..

[47] Sakamoto K., Karelina K., Obrietan K. (2011). CREB: A multifaceted regulator of neuronal plasticity and protection. J. Neurochem..

[48] Zhang Y., Smolen P., Alberini C.M., Baxter D.A., Byrne J.H. (2016). Computational model of a positive BDNF feedback loop in hippocampal neurons following inhibitory avoidance training. Learn Mem..

[49] Kaldun J.C., Sprecher S.G. (2019). Initiated by CREB: Resolving Gene Regulatory Programs in Learning and Memory: Switch in Cofactors and Transcription Regulators between Memory Consolidation and Maintenance Network. Bioessays.

[50] Palasz E., Wysocka A., Gasiorowska A., Chalimoniuk M., Niewiadomski W., Niewiadomska G. (2020). BDNF as a Promising Therapeutic Agent in Parkinson’s Disease. Int. J. Mol. Sci..

[51] Lisman J., Cooper K., Sehgal M., Silva A.J. (2018). Memory formation depends on both synapse-specific modifications of synaptic strength and cell-specific increases in excitability. Nat. Neurosci..

[52] Kandel E.R., Dudai Y., Mayford M.R. (2014). The Molecular and Systems Biology of Memory. Cell.

[53] Amidfar M., de Oliveira J., Kucharska E., Budni J., Kim Y.K. (2020). The role of CREB and BDNF in neurobiology and treatment of Alzheimer’s disease. Life Sci..

