# Supplementary material for: Copper Induces Cognitive Impairment in Mice via Modulation of Cuproptosis and CREB Signaling
Source: Nutrients. 2023 Feb 15;15(4):972. doi: 10.3390/nu15040972 (PMC9958748; doi:10.3390/nu15040972)
Supplement: Supplementary file 1 [file nutrients-15-00972-s001.zip › nutrients-2136055-supplementary.pdf]

Supplementary Materials for

Copper Induces Cognitive Impairment in Mice via Modulation of Cuproptosis and CREB Signaling

Zhang Ying, Zhou Qian, Lu Lu, Su Yu, Shi Wei, Zhang Hu, Liu Ran, Pu Yuepu and Yin Lihong*

*Corresponding author. Email: lhyin@seu.edu.cn

This file includes:

Supplementary Text Plasmid splicing sequence

Figure S1 Lentiviral vector of mouse CREB gene overexpression

Figure S2 Water intake of the mice

Figure S3 Copper intake of the mice

Supplementary Text

Plasmid splicing sequence

GCGATCGCCACCATGACCATGGAATCTGGAGCAGACAACCAGCAGAGTGGAG
ATGCTGCTGTAACAGAAGCTGAAAATCAACAAATGACAGTTCAAGCCCAGCCA
CAGATTGCCACATTAGCCCAGGTATCCATGCCAGCAGCTCATGCAACATCATCT
GCTCCCACTGTAACCTTAGTGCAGCTGCCCAATGGGCAGACAGTCCAGGTCCAT
GGCGTTATCCAGGCGGCCCAGCCATCAGTTATCCAGTCTCCACAAGTCCAAACA
GTTCAGATTTCAACTATTGCAGAAAGTGAAGATTCACAGGAGTCTGTGGATAGT
GTAACTGATTCCCAAAAACGAAGGGAAATCCTTTCAAGGAGGCCTTCCTACAG
GAAAATTTTGAATGACTTATCTTCTGATGCACCAGGGGTGCCAAGGATTGAAGA
AGAAAAGTCAGAAGAGGAGACTTCAGCCCCTGCCATCACCACTGTAACAGTGC
CAACCCCCATTTACCAAACTAGCAGTGGGCAGTACATTGCCATTACCCAGGGA
GGAGCAATACAGCTGGCTAACAATGGTACGGATGGGGTACAGGGCCTGCAGAC
ATTAACCATGACCAATGCAGCTGCCACTCAGCCGGGTACTACCATTCTACAGTA
TGCACAGACCACTGATGGACAGCAGATTCTAGTGCCCAGCAACCAAGTTGTTGT
TCAAGCTGCCTCAGGCGATGTACAAACATACCAGATCCGCACAGCACCCACGA
GCACCATTGCCCCTGGAGTTGTTATGGCGTCCTCCCCAGCACTTCCTACACAGCC
TGCTGAAGAAGCAGCACGGAAGAGAGAGGTCCGTCTAATGAAGAACAGGGAG
GCAGCAAGAGAATGTCGTAGAAAGAAGAAAGAATATGTGAAATGTTTAGAGA
ACAGAGTGGCAGTGCTTGAAAACCAAAACAAAACATTGATTGAGGAGCTAAA
AGCACTTAAGGACCTTTACTGCCACAAATCAGATGATTACAAAGATCACGACG
GGGATTATAAGGACCATGACATCGACTATAAGGATGATGACGACAAGTAAACG
CGT

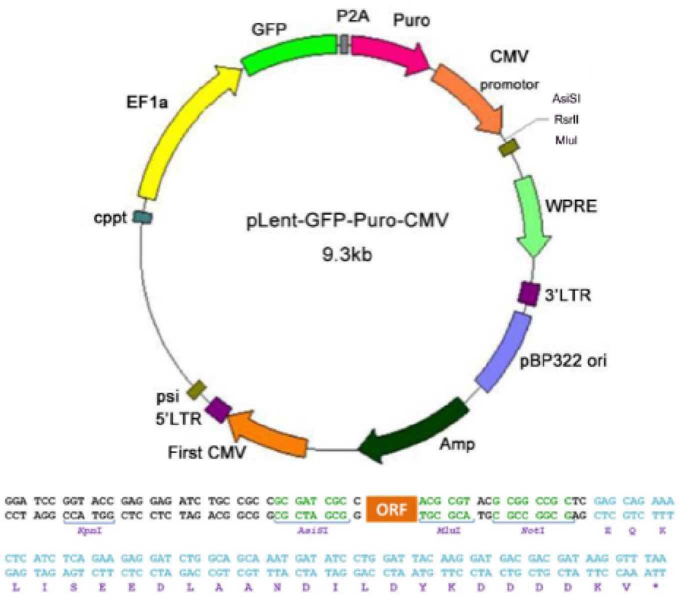

Figure S1 Lentiviral vector of mouse CREB gene overexpression

Restriction Enzyme cutting site is AsisI/MluI.

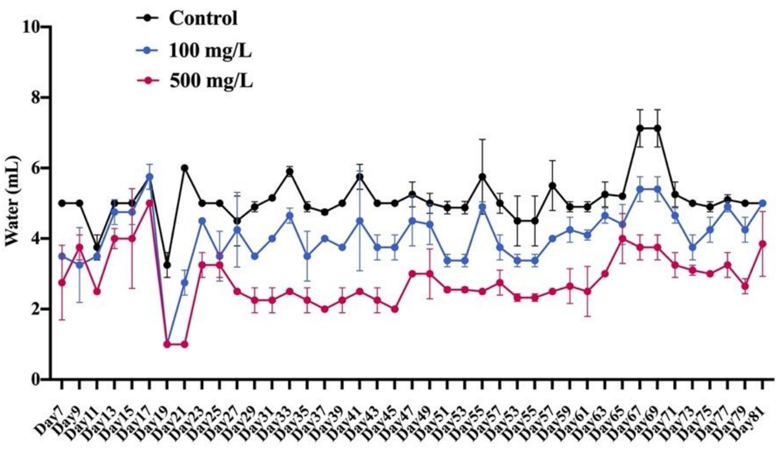

Figure S2 Water intake of the mice

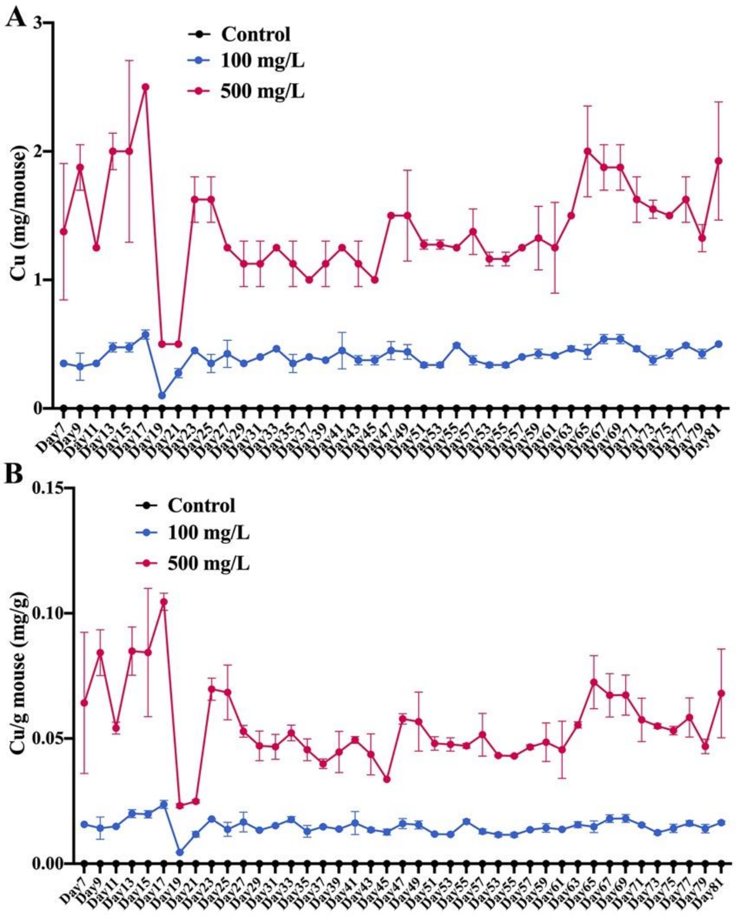

Figure S3 Copper intake of the mice

(A) Average copper intake per mouse. (B) Copper intake of unit weight mice.
